# Hydrogen Sulfide Prevents Synaptic Plasticity from VD-Induced Damage via Akt/GSK-3β Pathway and Notch Signaling Pathway in Rats

**DOI:** 10.1007/s12035-015-9324-x

**Published:** 2015-07-26

**Authors:** Chunhua Liu, Xiaxia Xu, Jing Gao, Tao Zhang, Zhuo Yang

**Affiliations:** 1 School of Medicine, State Key Laboratory of Medicinal Chemical Biology, Tianjin Key Laboratory of Tumor Microenvironment and Neurovascular Regulation, Nankai University, 94 Weijin Road, Tianjin, 300071 China; 2 College of Life Sciences, Nankai University, 94 Weijin Road, Tianjin, 300071 China

**Keywords:** Hydrogen sulfide, Vascular dementia, Long-term depression, Hippocampus

## Abstract

Our previous study has demonstrated that hydrogen sulfide (H_2_S) attenuates neuronal injury induced by vascular dementia (VD) in rats, but the mechanism is still poorly understood. In this study, we aimed to investigate whether the neuroprotection of H_2_S was associated with synaptic plasticity and try to interpret the potential underlying mechanisms. Adult male Wistar rats were suffered the ligation of bilateral common carotid arteries. At 24 h after surgery, rats were administered intraperitoneally with sodium hydrosulfide (NaHS, 5.6 mg·kg^−1^·day^−1^), a H_2_S donor, for 3 weeks in the VD+NaHS group and treated intraperitoneally with saline in the VD group respectively. Our results demonstrated that NaHS significantly decreased the level of glutamate. It obviously ameliorated cognitive flexibility as well as the spatial learning and memory abilities by Morris water maze. Moreover, NaHS significantly improved the long-term depression (LTD), and was able to elevate the expression of N-methyl-d-aspartate receptor subunit 2A, which plays a pivotal role in synaptic plasticity. Interestingly, NaHS decreased the phosphorylation of Akt, and it could maintain the activity of glycogen synthase kinase-3β (GSK-3β). Surprisingly, NaHS triggered the canonical Notch pathway by increasing expressions of Jagged-1 and Hes-1. These findings suggest that NaHS prevents synaptic plasticity from VD-induced damage partly via Akt/GSK-3β pathway and Notch signaling pathway.

Hydrogen sulfide modulated the ratio of NMDAR 2A/2B and improved the synaptic plasticity via Akt/GSK-3β pathway and Notch signaling pathway in VD rats.
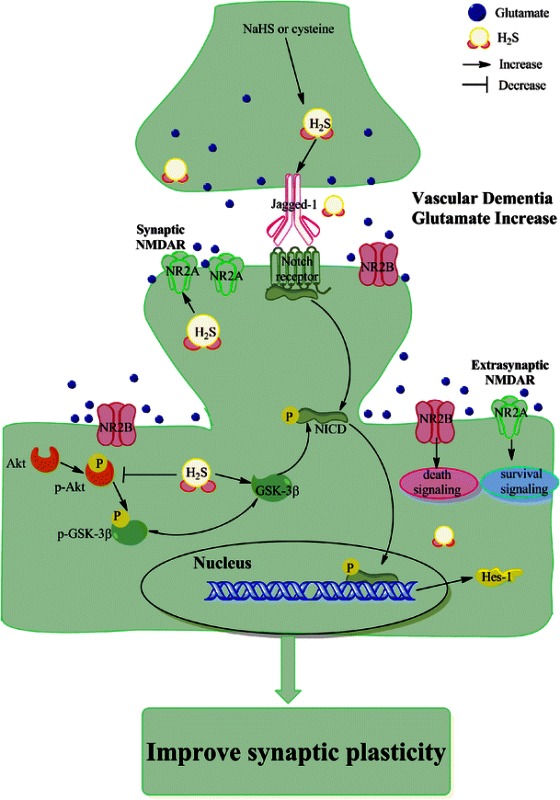

## Introduction

Epidemiological studies show that vascular dementia (VD) is the second commonest cause of dementia after Alzheimer’s disease (AD), and results from ischemic, ischemic-hypoxic, or hemorrhagic brain lesions [1–4]. The feature of VD is histopathological damage and progressive intellectual decline [5]. Unfortunately, the pathogenesis of the cognitive decline is still not fully understood, and there are fewer drugs licensed for the treatment of VD. The therapeutic strategy of VD is, so far, limited to control of known vascular risk factors in clinic [6].

As we all know, both long-term potentiation (LTP) and long-term depression (LTD) are two major forms of long-lasting synaptic plasticity in mammalian brain, and both of them are obviously characterized by a long-lasting increase or decrease in synaptic strength, respectively. LTP and LTD are considered to be related to the information storage, and therefore to learning and memory and other physiological processes. Glutamate, an important excitatory amino acid neurotransmitter, plays a pivotal role in the central nervous system [7, 8], and it acts on NMDARs (N-methyl-d-aspartate receptors ), AMPARs (α-amino-3- hydroxy-5-methylisoxazole-4-propionic acid receptors), KARs (kainate receptors), and mGluRs (metabotropic glutamate receptors) during the processes of LTP and LTD [9, 10]. However, high levels of glutamate is neurotoxic [11]. Studies show that the concentration of glutamate continues to be elevated in dementia and cerebral ischemia [7, 12], which leads to the excessive flux of calcium inside the neuron. Then, neuronal homeostasis is severely impaired and eventually, neurodegeneration happens [13]. Accumulating evidences indicate that the neurotoxic effects of glutamate appear to be mediated mainly via activation of NMDARs. Therefore, NMDARs appear to be a potential target for preventing progression of neurodegeneration. Theoretically, if agents are able to block pathological stimulation of NMDARs, they might be able to alleviate further neurodegeneration in VD [14]. And several promising results have been recently reported for uncompetitive NMDAR antagonists in the treatment of several forms of dementia [8, 14]. However, discouraging findings have begun to accumulate, and almost all of NMDAR antagonists of the clinical trials are terminated because of either lack of efficacy or their side effects [15]. Given the complex pathogenesis of dementia, it is necessary to develop more effective and safe agents.

Glycogen synthase kinase-3 (GSK-3) has recently been discovered to be a key regulator of synaptic plasticity [16]. GSK-3β activity is specifically involved in LTD mediated by NMDAR (NMDAR-LTD) [16]. The basal activity of GSK-3β is dependent on phosphorylation of Tyr216; on the contrary, the inhibition of GSK-3β is dependent on the phosphorylation of Ser9 and Ser389 [16, 17]. When GSK-3β activity is inhibited by phosphorylation at Ser9 by Akt, the NMDAR-LTD will be prevented [16]. In CNS, studies suggest that Notch signaling regulates neuronal synaptic plasticity, and therefore learning and memory [18]. There are Notch1, 2, 3, and 4 receptors of Notch signaling pathway [18]. Conditional deletion of Notch1 in postnatal hippocampus disrupts both LTP and LTD, and leads to deficits in learning and short-term memory [19]. When Notch signaling is activated, it will release Notch intracellular domain (NICD) [20]. Interestingly, Notch signaling pathway has crosstalk with other signaling pathways, such as Akt, Wnt, and NF-κB pathways [18]. Studies report that GSK-3β modulates Notch signaling via direct phosphorylation of NICD, and that the activity of GSK-3β protects NICD from proteasome degradation [21].

Hydrogen sulfide (H_2_S), as a notorious toxic gas, is recognized as the third gaseous signaling molecular along with nitric oxide (NO) and carbon monoxide (CO). It is synthesized endogenously from cysteine or homocysteine by cystathionine β-synthase (CBS) and cystathionine γ-lyase (CSE) [22]. The production of H_2_S is high in the brain. Moreover, H_2_S has been considered as a neuromodulator. A previous study demonstrate that H_2_S facilitates the induction of hippocampal LTP by enhancing the activity of NMDARs in neurons [23]. Our previous study has demonstrated that H_2_S improves LTP in hippocampus and inhibits the neuronal death induced by bilateral common carotid arteries ligation [24]. In addition, Zhang et al. have showed that H_2_S attenuates neuronal injury induced by VD via inhibiting apoptosis [25]. However, the mechanism of H_2_S on VD is poorly understood. In this study, we ligated bilateral common carotid artery to induce the rat model of VD, then evaluated the cognitive function by Morris water maze and LTD, and aimed to investigate whether the GSK-3β and Notch signaling pathway played an important role on H_2_S against VD.

## Materials and Methods

### Animal Care

Adult male 250–300 g Wistar rats were purchased from the Laboratory Animal Center, Academy of Military Medical Science of People’s Liberation Army. Rats were reared at specific pathogen-free condition under a 12-h light-dark cycle with lights on from 8 a.m. Food and water were freely available. All animal experiments were approved by the Animal Research Ethics Committee, School of Medicine, Nankai University. In addition, all animal experiments were performed in accordance with the Animal Management Rules of the Ministry of Health of the People’s Republic of China.

### Induction of VD

Rats were randomly assigned into three groups, i.e., sham group (*n* = 6), VD group (n = 8), and VD + sodium hydrosulfide (VD+NaHS) group (*n* = 8). The rat model of VD was established as previously described [24]. Briefly, rats were anesthetized by injecting 10 % chloral hydrate (35 mg·kg^−1^, i.p.), and were subjected to hair shaving before the surgical operation. Secondly, a longitudinal midline incision was made in the neck to expose the left and right common carotid artery. Finally, bilateral common carotid arteries were ligated with a 4–0 polypropylene in the VD and VD+NaHS groups. For the sham group, all operative procedures were performed identically, except for common carotid artery ligation. Rectal temperature was continually monitored and maintained at 36.5–37.5 °C with a heating blanket during VD surgery. Following surgery, rats were placed on a heating blanket until they recovered from the anesthesia, then they were placed in their cages and freely accessed food and water. At 24 h after surgery, the survival rate of the sham group was 100 %, and the survival rates of the VD and VD+NaHS groups were 75 %. Then, rats were injected intraperitoneally with NaHS (*n* = 6, 5.6 mg·kg^−1^·day^−1^, i.p.) daily for 3 weeks in the VD+NaHS group. And rats in the VD group were injected intraperitoneally with saline (*n* = 6, saline, i.p.).

### Morris Water Maze (MWM) Experiment

After 3 weeks post-injury, the spatial learning and memory ability of rats was measured by MWM as previously reported [26, 27]. The tank for MWM test was 150 cm in diameter and 60 cm in height and was filled with water to the depth of 45 cm. Nontoxic black ink was poured into water to make it dark. The tank was divided into four quadrants, namely zones 1, 2, 3 and 4. The MWM experiment comprised of four phases, i.e., initial training (IT), initial probe trials (IPT), reversal training (RT), and reversal probe trials (RPT). In IT stage, the 10-cm-diameter platform was submerged in the center of zone 1. Rats were trained every day with two sessions for 8-h interval. Each session consisted of four trials and each trial was performed for 60 s. Rats were placed into water at different quadrants in each session, respectively. They were allowed to swim freely until they stayed on the platform for at least 5 s. If failed to find the platform within 60 s, rats would be guided to the platform and stayed on it for 15 s. Both of the escape latency (time required to find the platform) and the swimming speed were recorded. On the fifth day, the IPT stage was performed. In this stage, the platform was removed. Rats were put into water and allowed to swim for 60 s, respectively. In this process, the platform crossings and quadrant dwell time were recorded. In RT stage, rats were trained every day with two sessions for 8-h interval. The platform was placed in the center of zone 4. Methods and parameters recorded were the same as those in IT stage. Both of the escape latency and the swimming speed were recorded. In RPT stage, the method was the same as that in IPT stage. Both of the quadrant dwell time and platform crossings were recorded.

### Long-Term Depression Recording

The long-term depression (LTD) recording was performed on rats after MWM experiment. LTD recording was performed as previously described [27, 28]. Rats were anesthetized by 30 % urethane anesthesia with a dosage of 4 ml/kg by intraperitoneal injection (Sigma-Aldrich, St. Louis, MO, USA) and positioned on a stereotaxic frame (SR-6N; Narishige, Japan). Recordings of the field excitatory post-synaptic potentials (fEPSPs) were made from the CA3 region (4.2 mm posterior to the bregma, 3.5 mm lateral to midline, 2.5 mm ventral below the dura) to the CA1 region (3.5 mm posterior to the bregma, 2.5 mm lateral to midline, 2.0 mm ventral below the dura). When the stable curves were obtained, baseline responses were recorded for 20 min. Then low-frequency stimulation (LFS) (900 pulses of 1 Hz for 15 min) was delivered to induce LTD. Following LFS, the amplitude of fEPSPs would be resumed for a further period of 60 min. After LTD recording, all rats were sacrificed. The hippocampus of the left brain was quickly removed at 4 °C and stocked at −70 °C. The right brain was quickly removed at 4 °C and immersed in 4 % paraformaldehyde fixed at 4 °C for 24 h, and then, they were dehydrated in sucrose solution and embedded with embedding medium of OCT at −20 °C for tissue section. The coronary slices (5 μm) were obtained and used for immunofluorescence staining of NMDAR2A (NR2A) and NMDAR2B (NR2B).

### Measurement of H_2_S

The concentration of H_2_S in hippocampus was determined as described previously [24]. Briefly, the hippocampus was homogenized in 50 mmol·l ^−1^ potassium phosphate buffer at 4 °C, pH 8.0 (12 % *w*·*v* ^−1^). The homogenate was centrifuged at 12,000 rpm for 10 min at 4 °C, and then 75 μl of supernatant was added to a 1.5-ml microtube containing 0.25 ml of 1 % zinc acetate. Thirdly, both of 20 mM of N, N-dimethyl-p-phenylendiammonium (NNDPD) in 7.2 M HCl and 30 mM FeCl_3_ in 1.2 M HCl were put into the microtube. The solution was then incubated at room temperature for 10 min. Next, 0.25 ml of 10 % TCA was added in order to remove proteins. Finally, the solution was centrifuged at 12,000 rpm for 10 min at 4 °C. The optical absorbance of the resulting solution was measured at 670 nm using a 96-well microplate reader. Each sample was measured in duplicate and the concentration of H_2_S was calculated against a standard calibration curve of NaHS (3.125–250 μM), which was performed on the same condition as the sample.

### Measurement of Glutamate

The levels of glutamate of hippocampus in all three groups were measured using the kit (Jiancheng, Nanjing, China) according to the manufacturer’s protocol.

### Western Blot Assay

The Western blot assay was performed as previously described [29]. Briefly, the rat hippocampus was homogenized in lysis buffer (Beyotime Biotechnology, Haimen, China) and centrifuged at 12,000 rpm for 10 min at 4 °C. The concentration of hippocampal protein was measured by using enhanced BCA protein assay kit (Beyotime Biotechnology, Haimen, China). The hippocampal protein lysates were separated on either 10 or 12 % SDS-PAGE gels, and then 50-μg proteins were transferred onto 0.22-μm polyvinylidene difluoride (PVDF) membrane (Promega Co. Ltd.) at 4 °C. Thirdly, the membrane was blocked in Tris-buffered saline with Tween-20 (TBST) containing 5 % skim milk for 1.5 h at room temperature. Fourthly, the PVDF membrane was incubated with primary antibody NR2A; NR2B; Hes-1; Jagged-1 (1:2000, Abcam, UK); GSK-3α/β; p-GSK-3β^ser9^; Akt; p-Akt^ser437^ (1:2000, Cell Signaling Technology, USA); CBS (Santa Cruz Biotechnology, USA); and β-actin (1:4000, Abcam, UK). Antibodies were diluted in TBST containing 5 % skim milk overnight at 4 °C. Finally, the second antibody (1:8000, Abcam, UK) was diluted in TBST. Protein band intensities were analyzed by using a Western blot detection system.

### Immunofluorescence Staining

According to the method described by Liu et al. [30], the immunofluorescence study was carried out as follows. Brain sections were washed in PBS for three times; subsequently, they were blocked with 10 % goat serum for 1 h at room temperature. Thirdly, brain sections were incubated with NR2A and NR2B (1:1000, Abcam, UK) for 18 h at 4 °C. Fourthly, they were washed by PBS for three times and incubated with Alexa 488-conjugated anti-rabbit IgG for 6 h at room temperature. Finally, they were stained with 4ʹ,6-diamidino-2-phenylindole (DAPI) for 3 min at room temperature and the fluorescent signals were detected by laser scanning confocal microscope.

### Statistical Analysis

Data were analyzed by SPSS 16.0 software. The results of escape latencies and swimming speeds in MWM test were analyzed by two-way repeated measures ANOVA followed by the Bonferroni multiple group comparison. The results of the platform crossings, quadrant dwell time, the temporal distribution of the quadrants, LTD recording, content of H_2_S and glutamate, and expression of proteins between three groups were performed using one-way ANOVA followed by a post hoc Turkey test. Data were presented as means ± S.E.M. and defined differences at *p* < 0.05 as statistically significant. All pictures were processed with Photoshop software.

## Results

### NaHS Decreased the Concentration of Glutamate, Elevated the Level of H_2_S, and the Expression of CBS in Hippocampus

**Fig. 1:**
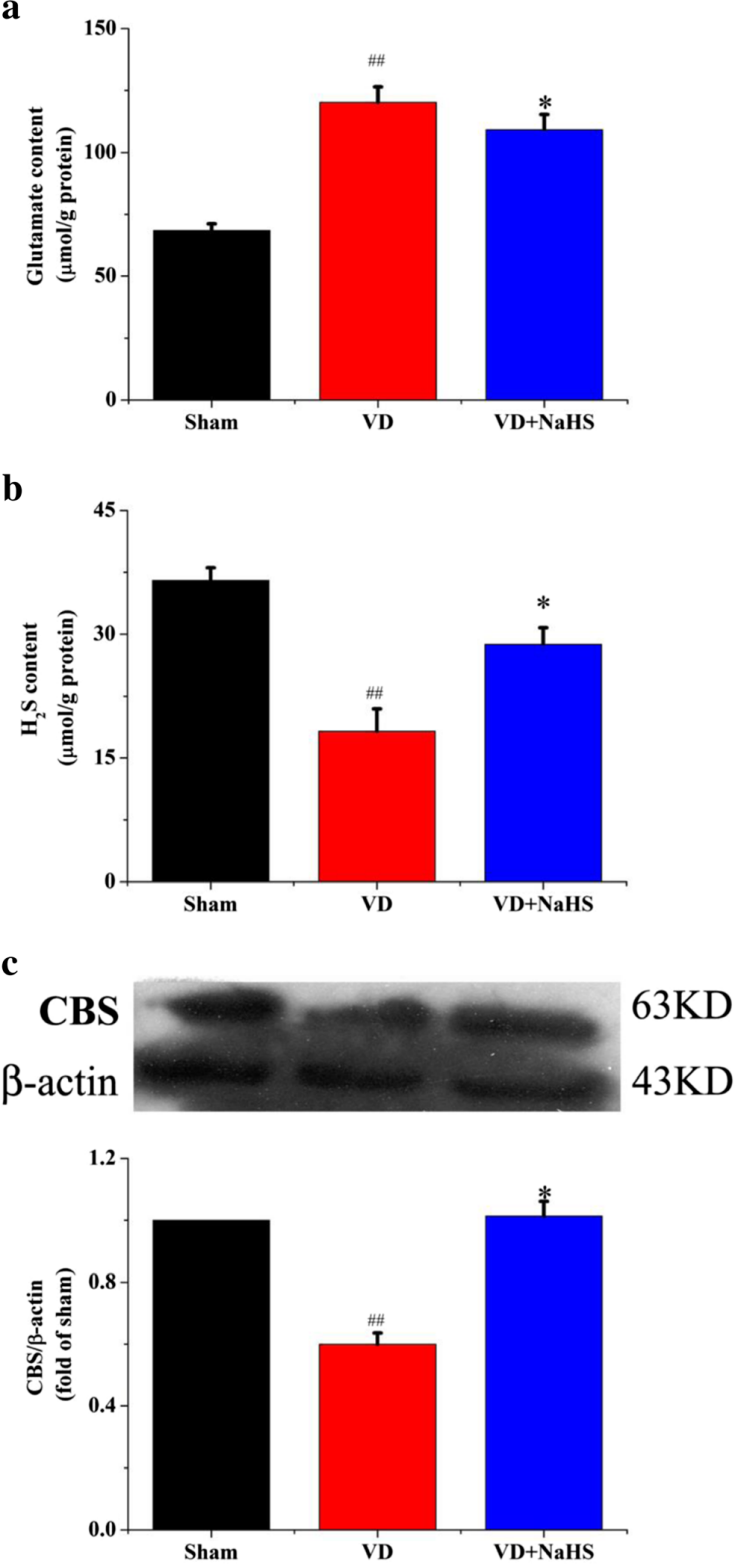
The contents of glutamate and H_2_S and the expression of CBS in hippocampus of the sham, VD, and VD+NaHS groups. **a** The content of glutamate. **b** The content of H_2_S. **c** Quantitative analysis of protein expression of CBS. Data are expressed as mean ± S.E.M. ^##^ *p* < 0.01 comparison between sham group vs. VD group. *p < 0.05, comparison between VD+NaHS vs. VD groups. *n* = 4 per group

Levels of H_2_S and glutamate in hippocampus were measured. Our data showed that the level of glutamate was obviously increased in hippocampus of the VD group compared with that of the sham group (*p* < 0.01, Fig. 1a). And the concentration of H_2_S was obviously decreased in the VD group. When administrated with NaHS, the concentration of glutamate in hippocampus was alleviated from 120.31 ± 6.11 to 109.24 ± 6.16 μmol·g^−1^ protein. Moreover, the content of H_2_S was significantly improved from 18.25 ± 2.72 to 28.75 ± 2.06 μmol·g^−1^ protein (*p* < 0.05, Fig. 1a, b). As shown in Fig. 1c, the expression of CBS was markedly decreased in the VD group, whereas NaHS remarkably improved the level of CBS (*p* < 0.01).

### NaHS Ameliorated the Impaired Spatial Learning and Memory Abilities

**Fig. 2:**
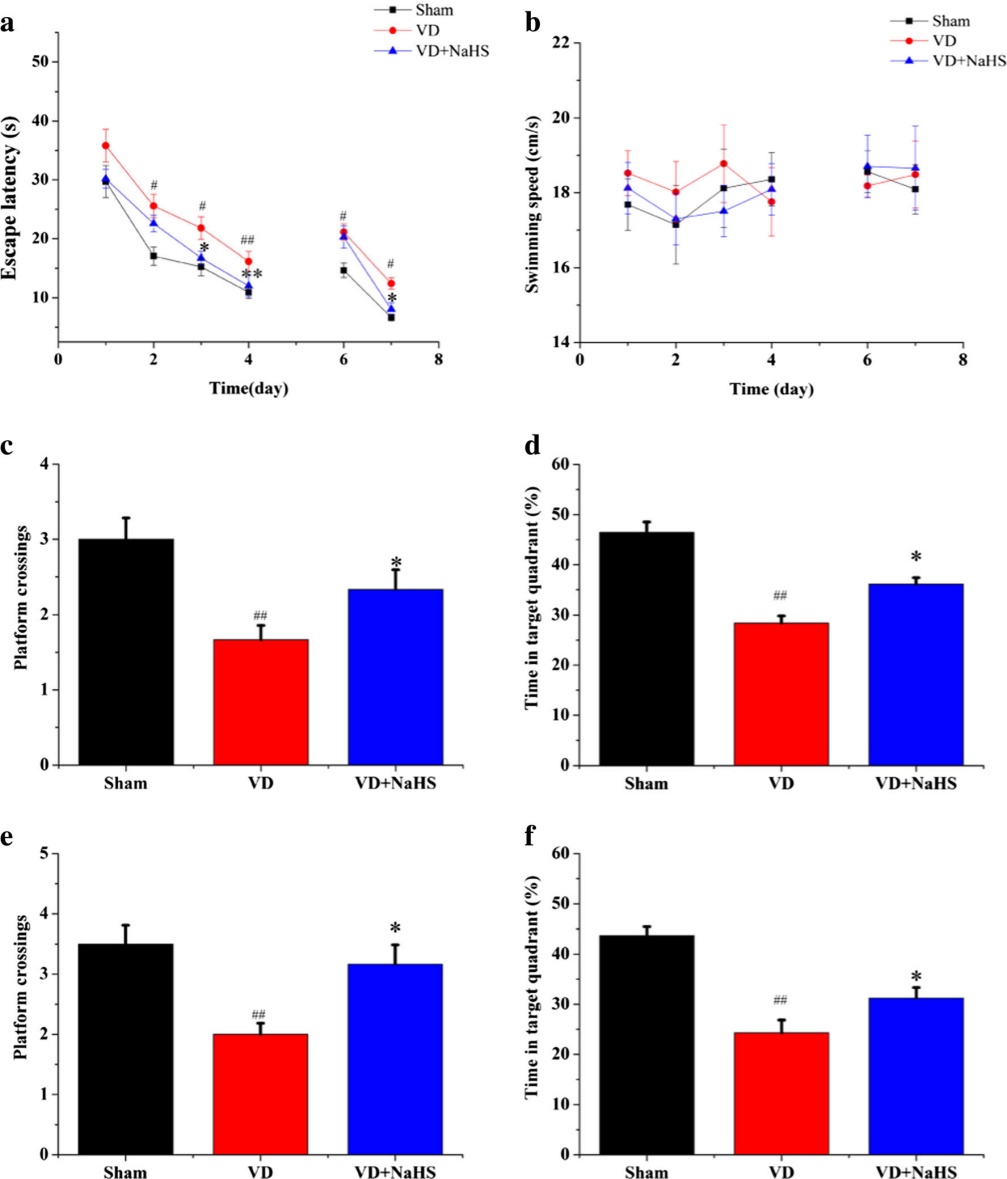
Measurement of spatial learning and reversal learning. **a** Escape latency calculated for each day in the sham, VD, and VD+NaHS groups on each training day in the IT and RT stages. **b** Swimming speed on each training day in the IT and RT stages. **c** Platform area crossings in the IPT stage within 1 min. **d** Percentage of time in the target quadrant in the IPT stage within 1 min. **e** Platform area crossings in RPT stage within 1 min. **f** Percentage of time in target quadrant in RPT stage within 1 min. Data are expressed as mean ± S.E.M. ^#^ *p* < 0.05, ^##^ *p* < 0.01 comparison between the sham vs. VD groups. *p < 0.05, **p < 0.01 comparison between the VD+NaHS vs. VD groups. *n* = 6 per group

In order to detect the effect of NaHS on spatial cognition, spatial learning and memory abilities were examined by MWM. Our data showed that the average escape latency was markedly decreased in all three groups during the IT stage without affecting swimming speeds (Fig. 2a, b), but rats in the VD group spent more time to find the platform compared with those in the sham group (*p* < 0.05, Fig. 2a). Interestingly, when administrated with NaHS, it was able to succeed in locating the platform much more quickly (*p* < 0.05, Fig. 2a). In the IPT stage, our results indicated that both the platform crossings and the quadrant dwell time were obviously decreased in the VD group (1.60 ± 0.21 and 28.39 ± 3.33 %, respectively, *p* < 0.01) (Fig. 2c, d), whereas NaHS remarkably improved both the platform crossings and the quadrant dwell time to 2.33 ± 0.30 and 36.11 ± 2.94 %, respectively (*p* < 0.05, Fig. 2c, d).

**Fig. 3:**
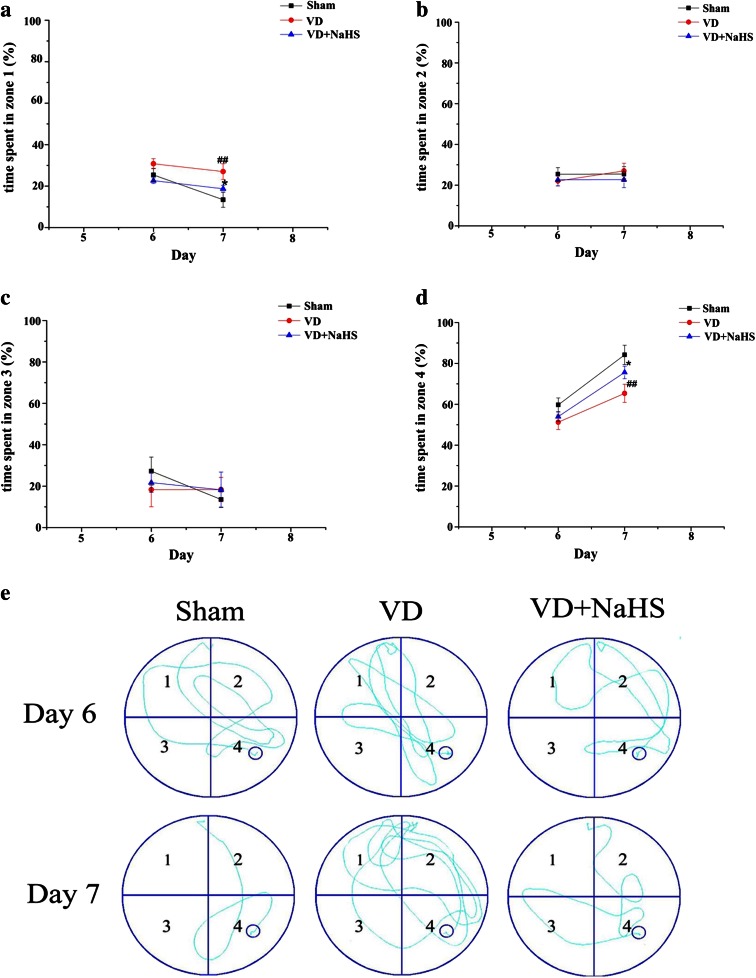
Comparison of temporal distribution in each quadrant of RT stage. **a** The temporal distribution in zone 1. **b** The temporal distribution in zone 2. **c** The temporal distribution in zone 3. **d** The temporal distribution in zone 4. Data are expressed as mean ± S.E.M. ^##^ *p* < 0.01 comparison between the sham vs. VD groups. **e** Representative swim traces of rats for each day in the sham, VD, and VD+NaHS groups. *p < 0.05 comparison between the VD+NaHS vs. VD group. *n* = 6 per group

The ability of reversal learning was performed to examine the effects of NaHS. During RT stage, the time of locating the hidden platform was markedly longer in the VD group than that of the sham group on days 6 and 7 (*p* < 0.05, Fig. 2a). Although NaHS failed to shorten the time of seeking the platform on the sixth day, it was able to reduce the average escape latency on the seventh day without affecting swimming speeds (*p* < 0.05, Fig. 2a, b). Other than that, it was found that both the platform crossings and the quadrant dwell time in RPT stage were obviously increased in the VD+NaHS group compared with those in the VD group (*p* < 0.05, Fig. 2e, f). In addition, the temporal distribution of the quadrants was analyzed in this study. When the hidden platform was located in zone 4 in the RT stage, rats of the VD group spent more time to seek the platform in zone 1 (original quadrant) compared with the sham group (*p* < 0.01, Fig. 3a) on the seventh day. In contrast, rats in the VD group spent less time in zone 4 (novel quadrant) compared with that of the sham group (*p* < 0.05, Fig. 3d). There was no significant difference in the time spent in zones 2 and 3 between the VD and the VD+NaHS groups (*p* > 0.05), but NaHS was able to decrease the time spending in zone 1 and improve the time spending in zone 4 on the seventh day (*p* < 0.05, Fig. 3a–d). The swim traces of all the three groups in the RT stage were showed in Fig. 3e. It can be seen that there was a short trajectory of reversal learning stage in both the sham and the VD+NaHS group, whereas the trajectory of the VD group was obviously longer (Fig. 3e). In addition, it was found that there were obviously more swimming trajectories in the original quadrant (zone 1) in the VD group compared to those in the VD+NaHS group. However, rats of the VD+NaHS group spent more time in the novel quadrant (zone 4) rather than other zones compared with the VD group.

### NaHS Ameliorated the Impaired Long-Term Depression in Hippocampus

**Fig. 4:**
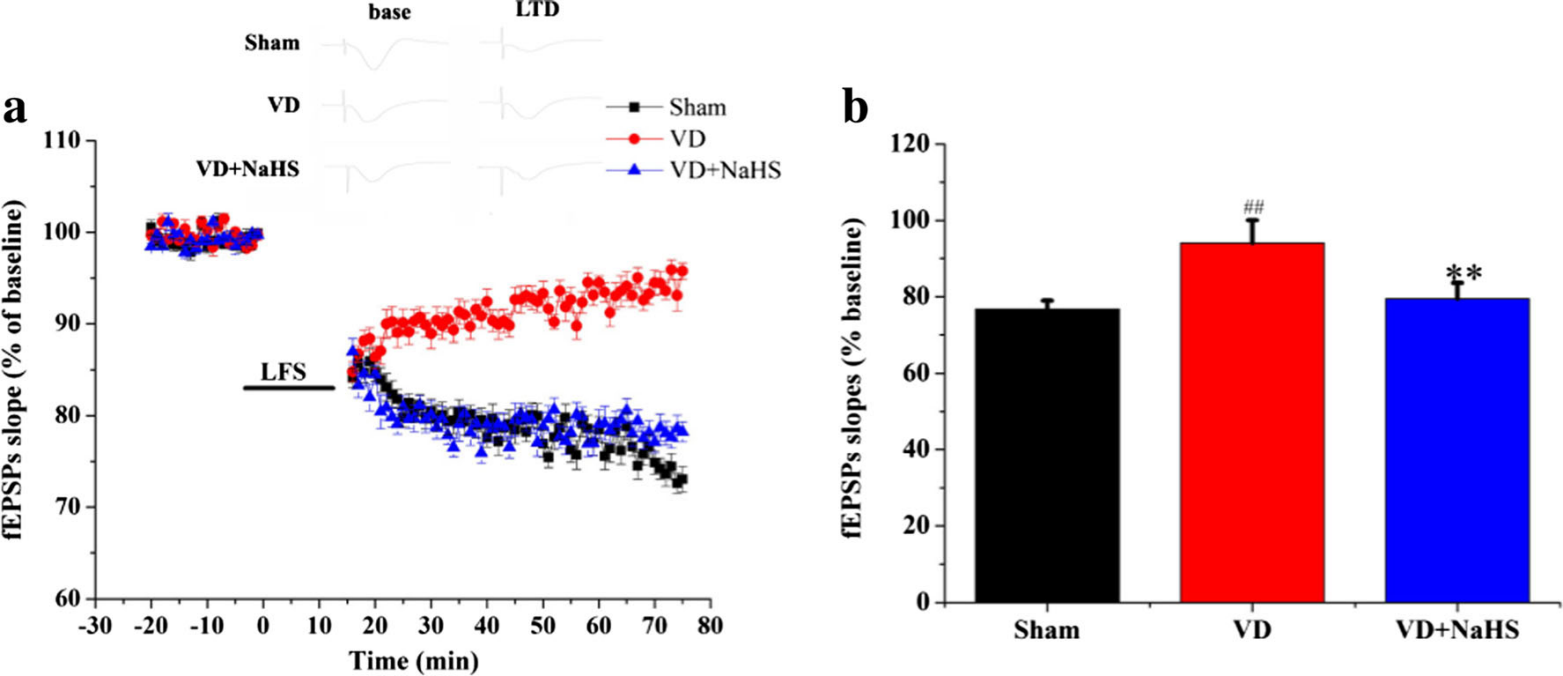
The effects of NaHS on LTD from CA3 to CA1 region of hippocampus. **a** Alterations in fEPSPs slopes after LFS in the sham, VD, and VD+NaHS groups. **b** Changes in fEPSPs slope in the sham, VD, and VD+NaHS groups. Data are expressed as mean ± S.E.M. ^##^ *p* < 0.01 comparison between the sham vs. VD groups. **p < 0.01 comparison between the VD+NaHS vs. VD groups. *n* = 6 for group

The LTD recording was performed to investigate the effects of NaHS on synaptic plasticity induced by VD. As shown in Fig. 4a, fEPSPs slopes were significantly decreased after the low-frequency stimulation from CA3 to CA1 regions and remained lower than the baseline in the sham group. And the fEPSPs slope was higher in the VD group, which indicated that LTD was suppressed in VD group compared with that of the sham group (*p* < 0.01, Fig. 4a, b). However, NaHS was able to reduce the fEPSPs slopes and maintained lower, which indicated that NaHS ameliorated the impaired synaptic plasticity induced by VD (*p* < 0.01, Fig. 4a, b).

### NaHS Modulated Cognitive Function-Associated Protein Expressions

**Fig. 5:**
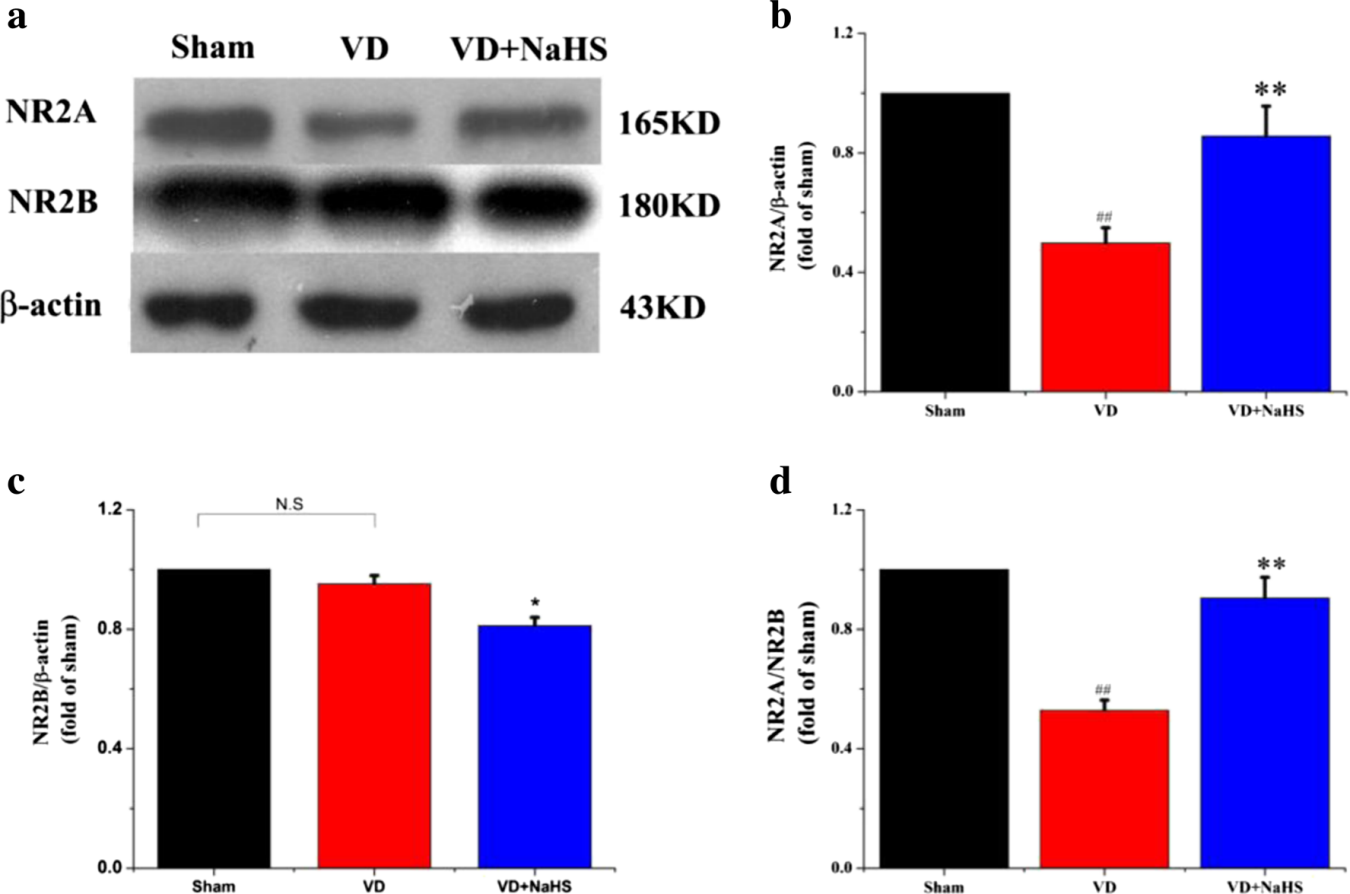
The expression of cognitive function associated protein detected by Western blot assay in hippocampus of the sham, VD, and VD+NaHS groups. **a** Quantitative analysis of protein expression of NR2A. **b** Quantitative analysis of protein expression of NR2B. **c** The ratio of NR2A/NR2B. Data are expressed as mean ± S.E.M. ^##^ *p* < 0.01 comparison between the sham vs. VD groups. **p < 0.01 comparison between the VD+NaHS vs. VD groups. *n* = 4 per group

**Fig. 6:**
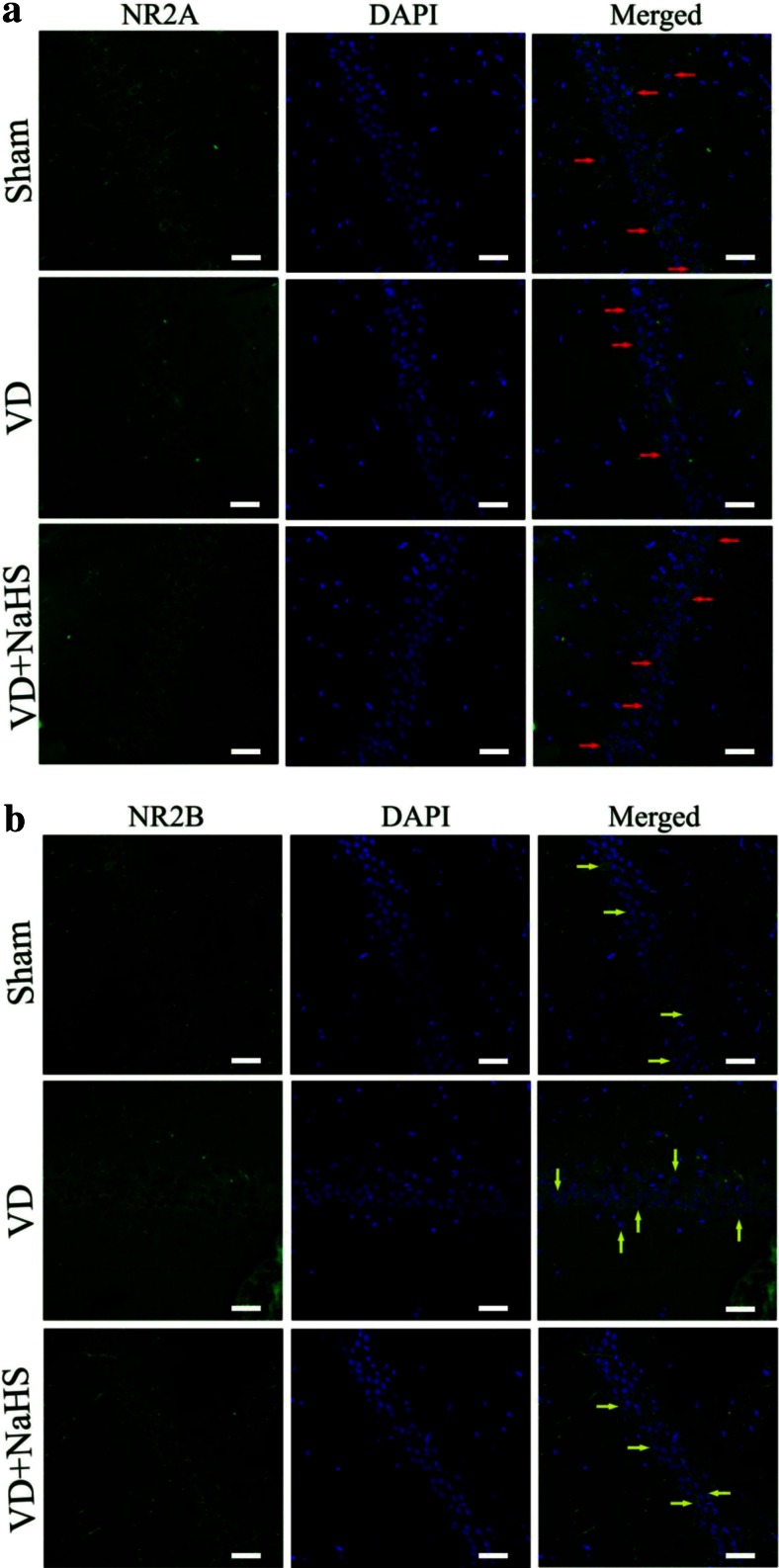
The expression of NR2A and NR2B detected by immunofluorescence staining (×400) in the CA1 region of hippocampus in the sham, VD, and VD+NaHS groups. Both of NR2A and NR2B were stained with green fluorescence by Alexa 488-conjugated anti-rabbit IgG. Nuclei of hippocampus were stained with blue fluorescence by DAPI. The protein expression of NR2A and NR2B were presented in the *left columns*, the morphology of nucleus was presented in the *middle columns*, and the merge pictures were presented in the *right columns*. Both NR2A and NR2B were presented by either *green point shape* distribution or a *green ring* around the nucleus in the *left and right columns. Red arrow* denotes NR2A expression in Fig. 6a and *yellow arrow* denotes NR2B expression Fig. 6b. *Scale bar* = 30 μm

The cognitive function-related proteins were examined by Western blot assay and immunofluorescence staining. Although there was no change of NR2B expression in hippocampus of the VD group (*p* > 0.05, Fig. 5a, c), NR2A expression was obviously downregulated to 0.50-fold compared with that of the sham group, then the ratio of NR2A/2B was decreased to 0.53 (*p* < 0.01, Fig. 5). Interestingly, NaHS was able to increase the level of NR2A to 0.84-fold and downregulated the expression of NR2B to 0.81-fold so as to improve the ratio of NR2A/2B to 0.91 (*p* < 0.01, Fig. 5). As shown in Fig. 6, both NR2A and NR2B were stained with green fluorescence by Alexa 488-conjugated anti-rabbit IgG. Nuclei in hippocampus were stained with blue fluorescence by DAPI. The protein expression of NR2A was presented by either green point shape distribution or a green ring around nucleus in the left and right columns in Fig. 6a. In the VD group, the expression of NR2A was obviously decreased compared with that of the sham group, which was consistent with the result of Western blot in Fig. 5. When treated with NaHS, it was markedly increased in expression of NR2A (Fig. 6a). On the other hand, there was no difference in the expression of NR2B between the three groups (Fig. 6b), which was consistent with the result of Western blot in Fig. 5.

### The Neuroprotective Mechanism of NaHS on VD

**Fig. 7:**
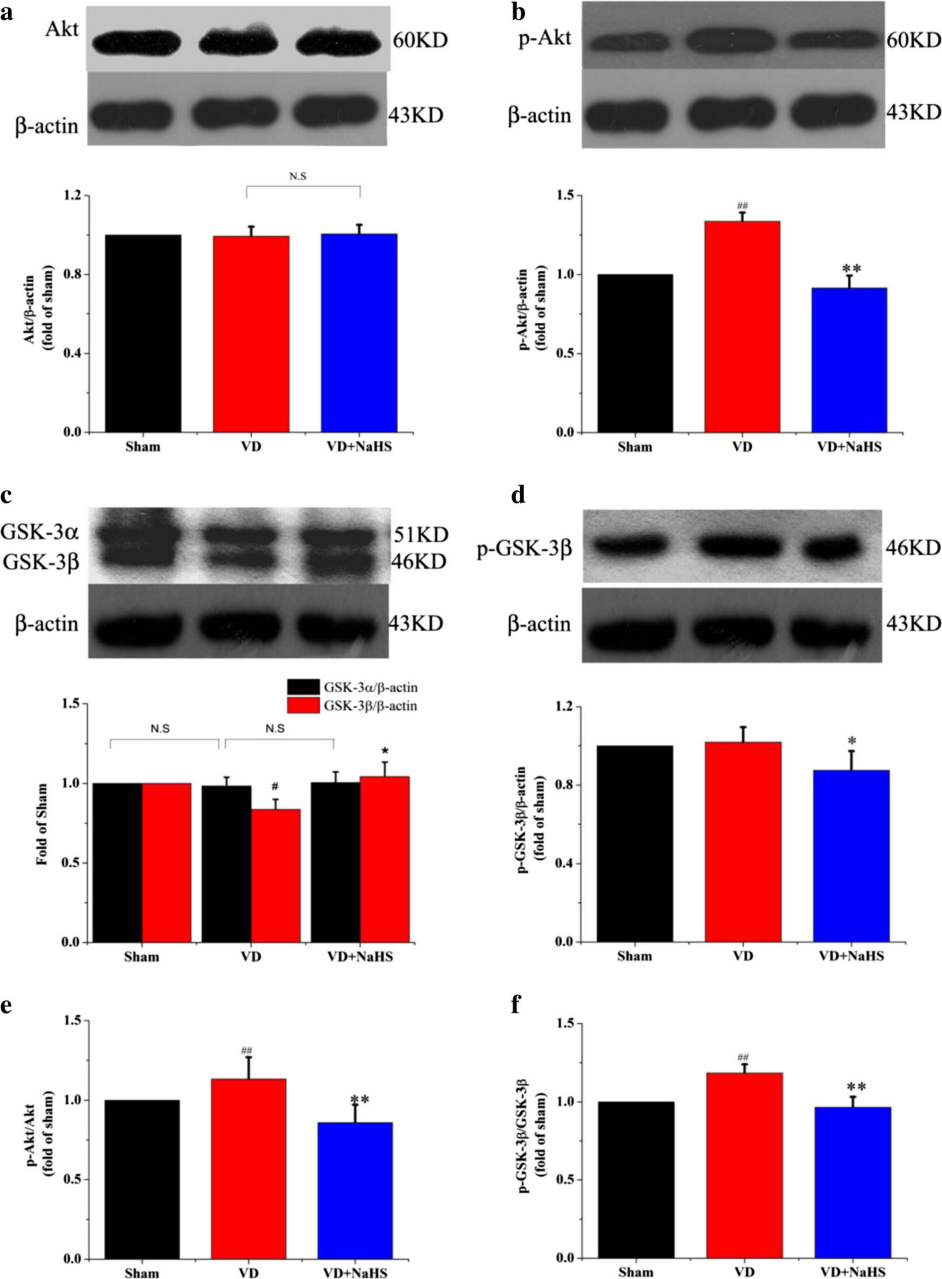
The expression of Akt, p-Akt, and GSK-3 detected by Western blot assay in hippocampus of sham, VD, and VD+NaHS groups. **a** Quantitative analysis of protein expression of Akt. **b** Quantitative analysis of protein expression of p-Akt. **c** Quantitative analysis of protein expression of GSK-3α/β. **d** Quantitative analysis of protein expression of p-GSK-3β. **e** Quantitative analysis of the ratio of p-Akt/Akt. **f** Quantitative analysis of the ratio of p-GSK-3β/GSK-3β. Data are expressed as mean ± S.E.M. ^#^ *p* < 0.05, ^##^ *p* < 0.01 comparison between the sham vs.VD groups. *p < 0.05, **p < 0.01 comparison between the VD+NaHS vs. VD groups. *n* = 4 per group

**Fig. 8:**
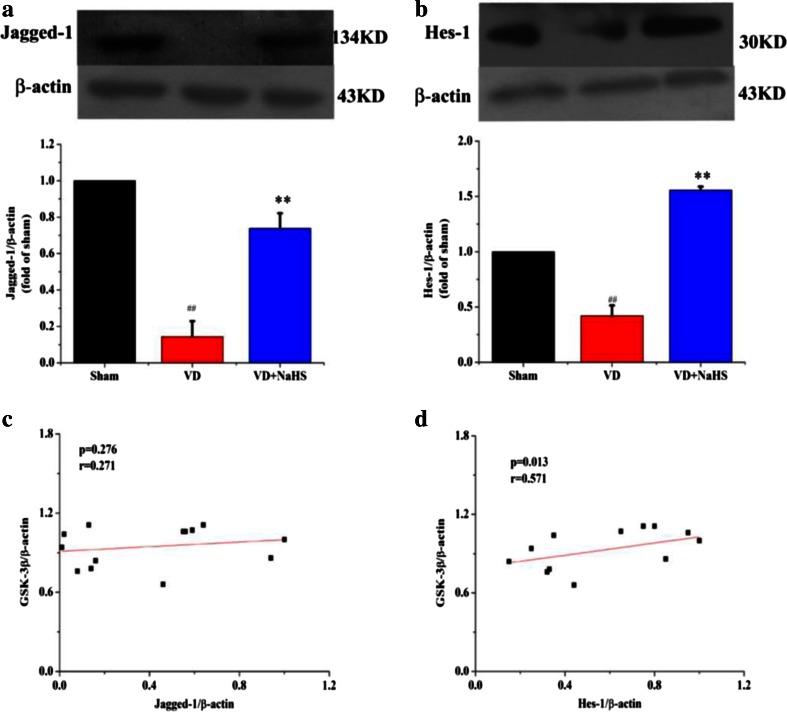
The expression of Jagged-1 and Hes-1 detected by Western blot assay in hippocampus of the sham, VD, and VD+NaHS groups. **a** Quantitative analysis of protein expression of Jagged-1. **b** Quantitative analysis of protein expression of Hes-1. **c** The correlation of Jagged-1 and GSK-3β. **d** The correlation of Hes-1 and GSK-3β. Data are expressed as mean ± S.E.M. ^##^ *p* < 0.01 comparison between the sham vs.VD groups. **p < 0.01 comparison between VD+NaHS vs. VD group. *n* = 4 per group

To investigate the signal transduction pathway of NaHS on VD, neuroprotective mechanism-related proteins were examined using Western blot assay. There was no significant difference in Akt expression between the three groups, but phosphorylated Akt (p-Akt) was markedly increased in the VD group (*p* < 0.05, Fig. 7a, b). Although NaHS failed to modulate the expression of Akt in hippocampus, it was able to reduce the p-Akt expression so as to decrease the ratio of p-Akt/Akt to 0.85 (*p* <0.05;, Fig. 7a–b, e). There was no difference in the expression of phosphorylated GSK-3β (p-GSK-3β) and GSK-3α between the VD and sham groups, but the expression of GSK-3β was obviously downregulated to 0.84 in the VD group, which increased the ratio of p-GSK-3β/GSK-3β to 1.18 (*p* < 0.05, Fig. 7c–d, f). However, NaHS was able to upregulate the expression of GSK-3β to 1.04, which was beneficial to reduce the ratio of p-GSK-3β/GSK-3β to 0.97 (*p* < 0.05, Fig. 7c, f). As shown in Fig. 8a–b, the expression of Hes-1 and Jagged-1 was significantly reduced in VD group (*p* < 0.01). Interestingly, NaHS was markedly alleviated levels of Hes-1 and Jagged-1 to 1.55- and 0.74-fold, respectively (*p* < 0.01). The linear relationship between the GSK-3β and the expression levels of Jagged-1 and Hes-1 proteins were also analyzed. In our study, the protein expression of Jagged-1 was not correlated with GSK-3β (*r* = 0.271, *p* = 0.276; Fig. 8c). However, the expression of Hes-1 was positively correlated with GSK-3β (*r* = 0.571, *p* = 0.013; Fig. 8d).

## Discussion

Accumulating evidences indicate that VD is the second commonest cause of dementia after Alzheimer’s disease (AD) [1]. However, the pathogenesis of VD is so far largely unknown and is likely multifactorial [1]. In our previous study, we reported that NaHS played a neuroprotective role on VD rats, but the underlying mechanism remained, so far, poorly understood.

Glutamate is an important excitatory amino acid neurotransmitter and plays a key role in the central nervous system [13]. However, high levels of glutamate show excitotoxicity, which is a critical mechanism contributing to neuronal death [31]. Several studies showed that the concentration of glutamate continued to be elevated in ischemia and dementia [12, 32, 33]. The high level of glutamate in hippocampus of VD rats was observed in our experiment, which indicated that VD was able to disturb the glutamate level of rats and led to excitotoxicity. When administrated with NaHS, the level of glutamate was significantly decreased. Although the glutamate level was decreased in hippocampus of VD rats by NaHS, it’s still hard to directly say that the neurotransmitter glutamate was changed according to our present data, which only suggested indirectly that the impairment of glutamate system could be alleviated by NaHS to some extent. Therefore, the underlying mechanism of NaHS on glutamate system still need to be further studied.

As known, H_2_S is mainly produced from l-cysteine (Cys) and homocysteine (Hcy) by CBS in the brain [34]. It was found that the plasma Hcy level of VD was significantly higher than that of normal control in clinical research, which suggested that the ability to transfer Hcy to H_2_S by CBS was damaged [35]. Zhang et al. have reported that the neuronal injury occurs with a decreasing number of neurons and an increasing apoptosis ratio in hippocampus after VD, while the H_2_S level is also decreased in plasma after VD [25]. Our previous study have reported that there is a clear edema and nuclear shrink phenomenon around pyramidal neurons in VD rats accompanied with decrease of H_2_S in VD rats, which is consistent with Zhang’s results [24, 25]. In this experiment, the level of H_2_S and expression of CBS were decreased in the VD group, which could be related to the apoptosis of neurons. When the death of neuron occurred, the capability to synthesize the protein of CBS could be damaged in neuron and therefore led to the decrease of the ability to generate H_2_S. Interestingly, NaHS could significantly attenuate neuronal death after VD [25]. Furthermore, studies have reported that the activation of NR2A is also beneficial to neuronal survival [36]. Our data showed that the expression of NR2A in VD+NaHS group was obviously upregulated compared with that of the VD group, and helped rats survive from neuronal death induced by VD. Thereafter, the synthesis and activity of CBS could be maintained in neurons, which could contribute to improving the production of H_2_S to some extent.

In our study, the MWM task was performed to evaluate the neuroprotection of NaHS. In the IT stage, NaHS was able to effectively alleviate cognitive deficiency to some extent, which was consistent with Li’s results [16]. Then, spatial reversal learning was carried out. On the second day of RT stage, rats in the VD group spent more time in zone 4 compared with other zones, but they obstinately searched the platform in zone 1 (the original quadrant). These results suggested that information storage was severely disrupted by VD. In contrast, rats in the VD+NaHS group could locate the platform much easier than that of the VD group, and they spent more time in zone 4 and less time in zone 1 than that of the VD group. It suggested that they developed a new strategy and explored the platform in the novel environment. Our results indicated that NaHS not only contributed to improve the spatial memory, but also ameliorate the deficits in cognitive flexibility.

As known, the LTD in hippocampus plays an important role in cognitive flexibility [28]. Actually, LTD could contribute to weaken previous memory traces; thus, in demand of a task change, it may prevent those traces from interfering with newly encoded information [28]. In this study, the capacity of behavioral flexibility partly weakened in the VD group. However, NaHS alleviated that deficiency to some extent. Subsequently, the LTD of hippocampus was recorded. Our results showed that VD induced the increase of fEPSP slope, which demonstrated that the synaptic plasticity was damaged. However, the impaired LTD was alleviated by the treatment of NaHS, which was consistent with the result of spatial reversal learning. Our data suggested that NaHS was able to prevent rats from the impairment of synaptic plasticity induced by VD.

Then, how does NaHS affect the synaptic plasticity? We inferred that NMDARs may be involved. Functional NMDARs are hetero-oligomeric proteins, which were composed of NR1 subunit and NR2 or NR3 subunits [37–39]. NR2A and NR2B were prevailing in adult cortex and hippocampus. Liu et al. showed that selectively blocking NR2B abolished the induction of LTD but not LTP. On the other hand, preferential inhibition of NR2A prevented the induction of LTP without affecting LTD production. By contrast, other studies found that inhibition of NR2A impaired LTP and also blocked LTD [24]. Thus, studies into the NMDAR subunit dependence of LTD produced even more contradictory results, making it hard to draw unifying conclusions [40]. In addition, NR2A is predominantly confined to synapses of mature neurons, while NR2B is distributed mainly extrasynaptically [37]. Studies both in vivo and in vitro reported that activation of either synaptic or extrasynaptic NR2B resulted in excitotoxicity and increasing neuronal apoptosis [36, 37]. However, the activation of either synaptic or extrasynaptic NR2A promoted neuronal survival and presented a neuroprotective action against both NMDA receptor-mediated and non-NMDA receptor-mediated neuronal damage [36]. Our data showed that the expression of NR2A was obviously decreased in hippocampus of VD rats, which suggested that the synaptic plasticity and capability of neuronal survival was markedly weakened in VD group. Surprisingly, the expression of NR2A was dramatically upregulated in the VD+NaHS group, which was beneficial to help rats prevent plasticity and survive from damage induced by VD. According to our results, we considered that NR2A subunits dominated over NR2B subunits with respect to both plasticity and neuronal survival on VD.

There are two isoforms of glycogen synthase kinase-3 (GSK-3), viz GSK-3α and GSK-3β, which is comprehensively expressed and involved in a variety of biological processes [41]. The high enzymatic activity is inhibited upon phosphorylation on residue ser9 of GSK-3β by Akt [42]. With regard to synaptic plasticity, GSK-3 appears to be essential for LTD, and the inhibition of GSK-3 prevents the induction of LTD [43]. In this experiment, VD induced a dramatic increase in the phosphorylation of Akt (p-Akt), which was consistent with Shu et al.’s results [44], and decreased the activity of GSK-3β. Fortunately, the expression of p-Akt was markedly reduced and maintained the activity of GSK-3β by NaHS. Therefore we inferred that NaHS modulated LTD partly via Akt/GSK-3β pathway.

In CNS, studies suggest that Notch signaling regulates neuronal plasticity, learning, and memory [18]. There are four Notch receptors, viz Notch1, 2, 3, and 4, and they are activated by specific ligands of the Jagged and Delta serrate member families. When ligands bind to Notch receptors, the proteolytic cleavage of Notch occurs. Subsequently, Notch receptors release the Notch intracellular domain (NICD). NICD is, then, translocated to nucleus to form a complex with the transcription factor CBF1/RBP-Jκ, which bind to their target genes such as Hes-1 and Hes-5 [20]. Interestingly, it appears that the reduction of Notch signaling affects sensory and memory processing [18]. Alberi et al. reported that conditional deletion of Notch1 in the postnatal hippocampus disrupted both LTP and LTD, and led to deficits in learning and short-term memory [19]. Our data showed that NaHS was able to ameliorate the decrease expression of Jagged-1. Moreover, the expression of Hes-1 was dramatically increased in the VD+NaHS group, suggesting a potential role of Notch on the neuroprotective effect of NaHS in VD rats’ brain. Our results implicated that NaHS modulated Notch signaling pathway by increasing not only its ligands but also its downstream target genes.

Although NaHS upregulated the expression of Jagged-1, it was still lower than that of the sham group. In contrast, the expression of Hes-1 was dramatically elevated, of which level was much higher than that of the sham group. Considering these results obtained, we speculated that NaHS enhanced the expression of Hes-1, possibly not only by upregulating ligands of Notch receptors. As known, Notch receptors modulate transcriptional targets following the proteolytic release of NICD, of which will be phosphorylated before translocating into the nucleus. Moreover, the phosphorylation of NICD has been identified within the nucleus and is imperative for translocating into the nucleus [45]. Studies report that GSK-3β modulates Notch signaling via direct phosphorylation of NICD, and that the activity of GSK-3β protects NICD from proteasome degradation [21]. In our present experiment, our data showed that NaHS not only improved the expression of GSK-3β, but also downregulated the phosphorylation of Akt in order to maintain the activity of GSK-3β. In addition, the expression of Hes-1 was positively correlated with GSK-3β, which suggested that GSK-3β could play an important role on the expression of Hes-1. In view of these results above, we speculated that NaHS protected NICD from proteasome degradation partly via Akt/GSK-3β pathway in order to elevate the expression of Hes-1.

In summary, we reported the neuroprotective effect of NaHS on VD rats. And our study demonstrated that NaHS was able to alleviate the excitotoxicity induced by VD through decreasing the level of glutamate, and elevate the expression of CBS to maintain the physical level of H_2_S. Moreover, NaHS reduced LTD to improve the performance of VD rats, which was confirmed by the spatial learning and memory abilities by MWM. Interestingly, NaHS reduced the phosphorylation of Akt and maintained the activity of GSK-3β, which was essential for LTD. Surprisingly, the canonical Notch signal pathway was triggered to ameliorate the impaired cognitive function in the VD+NaHS group. Thus, we conclude that NaHS prevents synaptic plasticity from VD-induced damage partly via Akt/GSK-3β pathway and Notch signaling pathway. Our investigation will shed new light on the mechanism of the neuroprotection of H_2_S on VD and might be used as a therapeutic method to cure neurodegenerative diseases.
